# Molecular Landscape of Therapy-related Myeloid Neoplasms in Patients Previously Treated for Gynecologic and Breast Cancers

**DOI:** 10.1097/HS9.0000000000000632

**Published:** 2021-08-18

**Authors:** Sabine Khalife-Hachem, Khalil Saleh, Florence Pasquier, Christophe Willekens, Anthony Tarabay, Leony Antoun, Thomas Grinda, Cristina Castilla-Llorente, Matthieu Duchmann, Cyril Quivoron, Nathalie Auger, Veronique Saada, Suzette Delaloge, Alexandra Leary, Aline Renneville, Ileana Antony-Debre, Filippo Rosselli, Stéphane De Botton, Flore Salviat, Christophe Marzac, Jean-Baptiste Micol

**Affiliations:** 1 Département d’Hématologie, Gustave Roussy, Université Paris-Saclay, Villejuif, France.; 2 INSERM U1287, Gustave Roussy, Université Paris-Saclay, Villejuif, France; 3 Hematology Laboratory, Hôpital Saint-Louis, Assistance Publique-Hôpitaux de Paris, University of Paris, France.; 4 Université de Paris, Génomes, biologie cellulaire et thérapeutique U944, INSERM, CNRS, Paris, France.; 5 Gustave Roussy, UMS AMMICA (UMS 3655), Villejuif, France; 6 Département de Biologie et Pathologie Médicales, Gustave Roussy, Université Paris-Saclay, Villejuif, France; 7 Département de Médecine Oncologique, Gustave Roussy, Université Paris-Saclay, Villejuif, France; 8 CNRS UMR9019, Gustave Roussy Institute, Université Paris-Saclay, Villejuif, France; 9 Gustave Roussy, Université Paris-Saclay, Service de Biostatistique et d’Epidémiologie, Villejuif, France

## Abstract

Definition of therapy-related myeloid neoplasms (TRMN) is only based on clinical history of exposure to leukemogenic therapy. No specific molecular classification combining therapy-related acute myeloid leukemia and therapy-related myelodysplastic syndromes has been proposed. We aimed to describe the molecular landscape of TRMN at diagnosis, among 77 patients with previous gynecologic and breast cancer with a dedicated next-generation sequencing panel covering 74 genes. We investigated the impact of clonal hematopoiesis of indeterminate potential-associated mutations (CHIP-AMs defined as presence at TRMN stage of mutations described in CHIP with a frequency >1%) on overall survival (OS) and the clinical relevance of a modified genetic ontogeny-based classifier that categorized patients in 3 subgroups. The most frequently mutated genes were *TP53* (31%), *DNMT3A* (19%), *IDH1/2* (13%), *NRAS* (13%), *TET2* (12%), *NPM1* (10%), *PPM1D* (9%), and *PTPN11* (9%). CHIP-AMs were detected in 66% of TRMN patients, with no impact on OS. Yet, patients with CHIP-AM were older and had a longer time interval between solid tumor diagnosis and TRMN. According to our modified ontogeny-based classifier, we observed that the patients with *TP53* or *PPM1D* mutations had more treatment lines and complex karyotypes, the “MDS-like” patients were older with more gene mutations, while patients with “De novo/pan-AML” mutations were younger with more balanced chromosomal translocations. Median OS within each subgroup was 7.5, 14.5, and 25.2 months, respectively, with statistically significant difference in multivariate analysis. These results support the integration of cytogenetic and molecular markers into the future TRMN classification to reflect the biological diversity of TRMN and its impact on outcomes.

## Introduction

Therapy-related myeloid neoplasms (TRMNs) arise after cytotoxic chemotherapy and/or radiotherapy administered for a prior neoplasm and include therapy-related acute myeloid leukemia (t-AML) and therapy-related myelodysplastic syndromes (t-MDS) as defined by 2016 WHO classification.^1^ TRMN occur in up to 2% of patients with malignancies and represent 10%–20% of all cases of MDS/AML.^2,3^ Different hypotheses have been proposed to explain the development of TRMN.^4,5^ Inherited predisposition, a rare event, or direct induction of fusion transcripts, well described for KMT2A-rearranged AML and acute promyelocytic leukemia (APL), can be responsible for TRMN. Recent evidence suggests that patient with clonal hematopoiesis of indeterminate potential (CHIP) at the time of treatment of their malignancy may have an increased risk of TRMN.^6^ CHIP is an age-associated genetic event characterized by one or more somatic mutations in hematopoietic stem cells (HSCs), including mutations in genes such as *DNMT3A*, *TET2*, *ASXL1*, and *TP53*. CHIP occurs in 10% of healthy individuals over 65 years. In patients with solid tumors, the prevalence of CHIP can rise up to 25%, mainly after chemotherapy exposure,^7^ and this is associated with a higher risk of primary hematological malignancies. It has been demonstrated in vitro and in vivo that *TP53* and *PPM1D* mutations confer a clonal advantage to mutated HSC after exposure to chemotherapy. This suggest the potential clonal selection that leads to TRMN in this specific context.^8,9^ Five-year overall survival (OS) rates of <10% are commonly reported in TRMN patients^10^; however, prognosis is mainly driven by cytogenetic and molecular findings: complex karyotype and *TP53* mutation-bearing TRMN are known to have a dismal prognosis,^11^ whereas therapy-related APL with t(15;17) can be cured without intensive chemotherapy.^12^ The current definition of TRMN is mainly based on chronological events and no molecular classification including t-AML and t-MDS together has been proposed, connecting physiopathology, patient characteristics and prognosis. Lindsley et al^13^ proposed an ontogeny-based classification for AML, which allows distinction of 3 genetic subgroups, a “*TP53* subgroup,” an “AML with MDS mutations,” and a “de novo/pan-AML” subgroup which appeared to be relevant in de novo but also t-AML.

In this setting, we aimed to define the molecular landscape of TRMN following treatment for gynecologic and breast cancers, and its impact on clinical outcome, as well as its relationship with the demographic, biological, and clinical features of the population studied. We searched for a suitable molecular classification of TRMN, especially focusing on “CHIP-associated mutations” (CHIP-AMs) and a modified genetic ontogeny-based classifier.

## Materials and methods

### Patients

Within our large single-center database (data protection approval, CNIL GR-2018-01), we identified 113 patients previously treated for breast or gynecologic cancers (the latter including any ovarian, endometrial, or cervical cancer) diagnosed with TRMN between January 2004 and December 2018 (see Supplemental Digital Table 1; http://links.lww.com/HS/A189). Patients with history of other hematological malignancies were excluded. We included patients only if they had a signed informed consent and available genomic DNA or viable cells collected at the time of TRMN diagnosis in our Center of Biological Resources (CRB). For deceased patients, we obtained an approval from the Comité de Protection des Personnes (CPP) Sud-Méditerranée II (Identification number: 2018A0264550/SI:18.09.27.62014) allowing the use of their data and materials.

In total, 77 patients fulfilled all inclusion criteria and were retained for the present analysis. For all these patients, we performed Next-Generation Sequencing (NGS) analysis using a 74-gene panel and the Haloplex technique (Agilent), followed by sequencing on a MiSeq instrument (Illumina) (Details in Supplemental Digital materials ; http://links.lww.com/HS/A189). In addition, *CEBPA*, *NPM1*, and *FLT3*-ITD mutations were screened by PCR and fragment analysis, as previously described.^14^ Moreover, paired samples of diagnostic bone marrow aspiration at the time of TRMN and peripheral blood at the time of primary cancer were available in 12 patients for NGS.

### TRMN classification

t-AML patients were classified into (favorable/intermediate/adverse) risk groups according to the ELN 2017 classification,^15^ and t-MDS patients were classified according to IPSS score into low-risk, intermediate 1/intermediate 2, and high-risk groups.^16^ According to DNA sequencing results, patients were then classified into “CHIP-AM” category if they had any of the genes described with a frequency > 1% in first CHIP papers^17,18^ (Supplemental Digital Table 2; http://links.lww.com/HS/A189) or “no-CHIP” category, if none of these mutations were detected. The most commonly mutated genes in CHIP, considered to be CHIP-AM in our study, are *DNMT3A* (52.4%), *TET2* (9.1%), *ASXL1* (8.6%), *JAK2* (4.9%), *PPM1D* (4.6%), *SF3B1* (3.5%), *TP53* (3.3%), *SRSF2* (1.6%), and *CBL* (1.3%).” Patients were also classified into 3 subgroups according to a modified genetic ontogeny-based classifier^13^: “*TP53/PPM1D*” subgroup, “MDS-like” subgroup, and “*de novo/pan*-AML” subgroup. The “MDS-like” subgroup is defined by the presence of *SRSF2, SF3B1, U2AF1, ZRSR2, ASXL1, EZH2, BCOR*, *or STAG2* mutations (these mutations were defined as highly specific to post-MDS AML^13^), or MDS disease not included in the 2 other groups. “*De novo/pan*-AML” included t-AML patients without *“TP53/PPM1D*” or “MDS-like.” Moreover, cytogenetic alterations were classified as failure, normal karyotype, complex karyotype (presence of 3 or more aberrations), and balanced translocation (including KMT2A rearrangements, core binding factor translocations, and APL).

### Statistical analysis

Clinical, pathological, cytogenetic data, and information regarding treatment and outcomes were collected from the patient’s medical records.

The type of first cancer treatment was categorized as chemotherapy alone, radiotherapy alone or chemo/radiotherapy. The type of TRMN treatment was categorized as best supportive care, low-intensity treatment (low-dose cytarabine and hypomethylating agents), intensive treatment (including induction chemotherapy and allogeneic hematopoietic stem cell transplantation [HSCT]).

Time interval between solid tumor diagnosis and TRMN was calculated as time from the date of primary cancer diagnosis to the date of TRMN diagnosis. OS was calculated from the date of TRMN diagnosis to the date of death from any cause or censored at the last follow-up. Event-free survival (EFS) was defined as time from diagnosis to induction failure, relapse, or death from any cause. Database cutoff December 31, 2019 (1 y after the last patient inclusion). Statistical analyses were performed with R software version 3.6.1. The comparison of percentages was carried out with a Pearson’s Chi-square test or a Fisher’s exact test. The distributions of a quantitative variable according to the modalities of a qualitative variable were compared with a Mann–Whitney test. The distributions of survival data were estimated using the Kaplan–Meier method, compared with the log-rank test and hazard ratios (HRs) with 95% confidence intervals (95% CIs). To identify variables associated with OS, a Cox proportional hazards regression analysis of candidate prognostic factors was performed. All the tests were two-sided and considered to be significant when *P* <0.05.

## Results

### Patient characteristics

Seventy-seven patients were identified between April 2004 and December 2018 of whom 49 (64%) t-AML and 28 (36%) t-MDS. Median time between primary cancer and TRMN was 5.1 years (95% CI, 3.6–6.6). Primary cancers were breast (n = 54, 70%), ovarian (n = 18, 23%), endometrial (n = 3, 4%), and cervical (n = 2, 3%) cancers. Patients were treated with radiotherapy alone (n = 10, 13%), cytotoxic agent alone (n = 15, 19%), or cytotoxic agent and radiotherapy (n = 52, 68%). They received a median of one treatment line (interquartile range [IQR], 1–3), but 20 patients (26%) received more than 2 treatment lines. At TRMN diagnosis, median age was 62 years old (IQR, 54–70) and WHO performans status was 0 or 1 for 56 patients (73%). Karyotype was a failure in 4 (5%) patients, normal in 15 patients (20%), and complex in 25 patients (32%). Ten (13%), 4 (5 %), 5 (7%), and 14 (18%) patients harbored genetic alterations characteristic of KMT2A-rearranged, core binding factor, APL leukemia, and other cytogenetic abnormalities, respectively (Table 1, Supplemental Digital Table 3; http://links.lww.com/HS/A189).

**Table 1.** Patients Characteristics According to CHIP-AM Mutation.

	CHIP-AM	No-CHIP	Total	*P*
	n = 51	n = 26	n = 77	
First cancer, n (%)				0.1^*a*^
Breast cancer	33 (65%)	21 (81%)	54 (70%)	
Gynecological cancer	18 (35%)	5 (19%)	23 (30%)	
First cancer treatment, n (%)				0.2^*a*^
Chemotherapy/radiotherapy	36 (71%)	16 (62%)	52 (68%)	
Radiotherapy alone	4 (8%)	6 (23%)	10 (13%)	
Chemotherapy alone	11 (22%)	4 (15%)	15 (19%)	
Number of treatment lines, mean (SD)	2.33 (1.97)	1.65 (1.09)	2.1 (1.74)	0.2^*b*^
Alkylating agent, n (%)	42 (82%)	21 (81%)	63 (82%)	0.9^*a*^
Anthracycline, n (%)	29 (57%)	20 (77%)	49 (64%)	0.08^*a*^
Median time between TRMN	6.6 (3–13.2)	2.9 (2–4.8)	5.1 (3.6–6.6)	<0.001^*c*^
and cancer, y (95% CI)				
Median age at TRMN, y (IQR)	64 (56–72.5)	56 (49.25–65.75)	62 (54–70)	0.022^*b*^
Performance status, n (%)				0.6^*a*^
0–1	36 (71%)	20 (77%)	56 (73%)	
2–3	15 (29%)	6 (23%)	21 (27%)	
Myeloid neoplasm, n (%)				0.2^*a*^
AML	30 (59%)	19 (73%)	49 (64%)	
MDS	21 (41%)	7 (27%)	28 (36%)	
Cytogenetic failure, n (%)	3 (6%)	1 (4%)	4 (5%)	>0.99^*d*^
Normal karyotype, n (%)	9 (18%)	6 (23%)	15 (21%)	>0.99^*a*^
Balanced translocation, n (%)	6 (12%)	13 (50%)	19 (25%)	<0.001^*a*^
Complex karyotype, n (%)	24 (47%)	1 (4%)	25 (34%)	<0.001^*a*^
Gene mutations, mean (SD)	2.67 (1.53)	1.42(1.17)	2.25 (1.53)	<0.001^*b*^
ELN 2017 AML classification, n (%)				0.009^*a*^
Adverse	18 (60%)	3 (16%)	21 (43%)	
Favorable	7 (23%)	8 (42%)	15 (31%)	
Intermediate	5 (17%)	8 (42%)	13 (27%)	
MDS IPSS score, n (%)				0.038^*d*^
Low/Int1	1 (5%)	3 (43%)	4 (14%)	
Int2/high	20 (95%)	4 (57%)	24 (86%)	
TRMN treatment, n (%)				0.2^*a*^
Intensive	19 (37%)	15 (58%)	34 (44%)	
Low dose	20 (39%)	7 (27%)	27 (35%)	
BSC	12 (24%)	4 (15%)	16 (21%)	
CR rate, n (%)	25 (49%)	17 (65%)	42 (55%)	0.2^*a*^
HSCT, n (%)	10 (20%)	6 (23%)	16 (21%)	0.7^*a*^
Median OS, mo (95% CI)	14.3 (8.7–23)	12.8 (8.6–NR)	13.9 (10.5–20.4)	0.4^*c*^
Median EFS, mo (95% CI)	9.6 (6.1–17)	10.6 (5.7–34.6)	10.1 (6.8–16.3)	0.4^*c*^

^*a*^Pearson's Chi-square test.

^*b*^Wilcoxon test.

^*c*^Logrank test.

^*d*^Fisher's exact test.

AML = acute myeloid leukemia; BSC = best supportive care; CHIP-AM = CHIP-associated mutations; CI = confidence interval; CR = complete remission; EFS = event-free survival; HSCT = hematopoietic stem cell transplantation; IQR = intervalle quartile range; MDS = myelodysplastic syndrome; NR = not reached; OS = overall survival; TRMN = therapy-related myeloid neoplasm.

### Molecular landscape at TRMN stage

**Figure 1.**
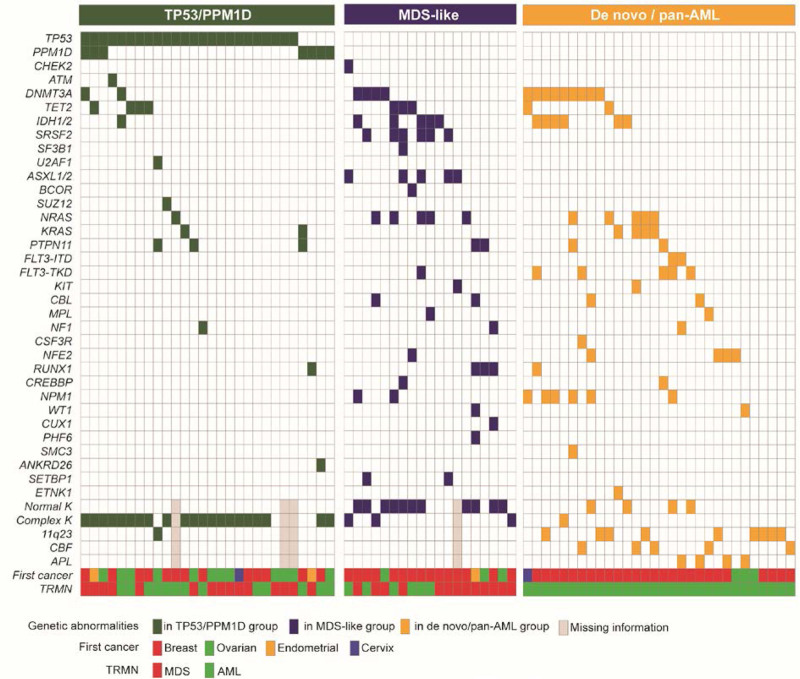
**Commutation plot of the 77 TRMN patients.** Mutations are depicted by colored bars, and each column represents 1 of the 77 sequenced subjects. Colors reflect modified genetic ontogeny-based classifier groups described by Lindsley et al.

The most frequently mutated genes at TRMN diagnosis were: *TP53* (n = 24, 31%), *DNMT3A* (n = 15, 19%)*, IDH1/2* (n = 11, 13%)*, NRAS* (n = 11, 13%)*, TET2* (n = 9, 12%)*, NPM1* (n = 8, 10%)*, PPM1D* (n = 7, 9%), and *PTPN11* (n = 7, 9%) (Figure 1, Supplemental Digital Table 3; http://links.lww.com/HS/A189). Patients had a median of 2 mutations (IQR, 1–3). No gene mutation was identified in 7 (10%) patients. According to 2017 ELN risk stratification, genetic risk for t-AML was favorable, intermediate and adverse in 15 (30%), 13 (27%), and 21 (43%), respectively. According to IPSS score, 24 (86%) of the t-MDS patients were classified as high risk/intermediate 2 and 4 (14%) as intermediate 1/low risk.

It has been suggested that TRMN could emerge by clonal selection.^4^ Based on literature data, we classified patients as TRMN with CHIP-AMs or not (Table 1, Supplemental Digital Figure 1; http://links.lww.com/HS/A189) according to the presence of mutations usually found in patients with CHIP with a frequency >1%. CHIP-AMs were detected in 51 patients (66%).

To give substance to this classification we performed NGS on 12-paired samples. Nine of these patients had a CHIP-AM at TRMN stage (Supplemental Digital Table 4; http://links.lww.com/HS/A189). As expected, no CHIP-AM was detected at cancer stage for the 3 patients without CHIP-AM at TRMN stage. In 7 out of the others 9 patients (78%), at least one of the CHIP-AM was detected as preleukaemic clonal hemopoiesis at the cancer stage.

Interestingly, the median age at TRMN diagnosis in patients with CHIP-AM versus patients with no-CHIP was higher (64 [IQR, 56–72.5] versus 56 [IQR, 49.25–65.75] y old, *P* = 0.022), the median time interval between cancer diagnosis and TRMN was longer (6.6 y [IQR, 3–13.2] versus 2.9 [IQR, 2–4.8], *P* < 0.001). The number of treatment lines and types of chemotherapy for the previous cancer did not impact the emergence of CHIP-AM, except for a trend toward less anthracycline used in the CHIP-AM group (57%, versus 77%, *P* = 0.08). CHIP-AM TRMN had more complex karyotypes (47% versus 4%, *P* < 0.001), and conversely fewer balanced translocations (12% versus 50%, *P* < 0.001). Accordingly, t-AML with CHIP-AM was more often classified as adverse (60% versus 16%, *P* = 0.009) and t-MDS CHIP-AM more often classified as intermediate 2/high risk, (95% versus 57%, *P* = 0.038).

We decided to validate in our cohort of TRMN a modified genetic ontogeny-based classifier proposed by Lindsley et al^13^ that classifies AML based on genetic ontogeny rather than clinical ontogeny assignment. Twenty-eight (36%), 19 (25%) and 30 (39%) patients belonged to the “TP53/PPM1D,” “MDS-like,” and “de novo/pan-AML” subgroups. Patient characteristic are described in Table 2. Interestingly, patients from “de novo/pan-AML” group were younger (56 y old [IQR, 50.25–64], *P* = 0.004), had more frequently a history of breast cancer (87%, *P* = 0.002), and fewer treatment lines for the first cancer (mean 1.63, SD [1.03], *P* = 0.05) but were more often treated with anthracycline (80%, *P* = 0.003). They displayed t-AML (100% versus 0% t-MDS, *P* < 0.0001) with more balanced translocation karyotypes (60%, *P* < 0.0001). Conversely, “TP53/PPM1D” group had more often a history of gynecological cancer (54%, *P* = 0.002), more treatment lines (mean 2.79, SD [2.22], *P* = 0.05), more MDS (54%, *P* < 0.0001) and more complex karyotypes (84%, *P* < 0.0001). Finally, patients in the “MDS-like” group were older (68 y old, IQR [61.5–76.5]) with more frequently an MDS phenotype (68%, *P* < 0.0001), normal karyotype (61%, *P* < 0.0001) and a trend toward a higher number of gene mutations (64% had ≥3 mutations, *P* = 0.06).

**Table 2.** Patients Characteristics According to Lindsley’s Modified Genetic Classifier.

	Lindsley’s Modified Genetic Classifier			Total	*P*
	TP53/PPM1D	MDS-like	De novo-like		
First cancer, n (%)					0.002
Breast cancer	13 (46%)	15 (79%)	26 (87%)	54 (70%)	
Gynecological cancer	15 (54%)	4 (21%)	4 (13%)	23 (30%)	
First cancer treatment, n (%)					0.1
Chemotherapy/radiotherapy	16 (57%)	14 (74%)	22 (73%)	52 (68%)	
Radiotherapy alone	2 (7%)	3 (16%)	5 (17%)	10 (13%)	
Chemotherapy alone	10 (36%)	2 (11%)	3 (10%)	15 (19%)	
Median treatment lines, Moy (std)	2.79 (2.22)	1.84 (1.61)	1.63 (1.03)	2.1 (1.74)	0.05^*a*^
Alkylating agent, n (%)	23 (82%)	15 (79%)	25 (83%)	63 (82%)	0.9
Anthracycline, n (%)	11 (39%)	14 (74%)	24 (80%)	49 (64%)	0.003
Median time between TRMN and cancer, y (95% CI)	6.15 (5–10.3)	4.4 (3.3–14.4)	4.3 (2.7–8.4)	5.1 (3.6–6.6)	0.4^*b*^
Median age at TRMN, y (IQR)	65.5 (56.5–74)	68 (61.5–76.5)	56 (50.25–64)	62 (54–70)	0.004^*a*^
Performance status, n (%)					0.1
0–1	17 (60%)	16 (84%)	23 (77%)	56 (73%)	
2–3	11 (39%)	3 (16%)	7 (23%)	21 (27%)	
Myeloid neoplasm, n (%)					<0.0001
AML	13 (46%)	6 (32%)	30 (100%)	49 (64%)	
MDS	15 (54%)	13 (68%)	0 (0%)	28 (36%)	
Cytogenetic failure, n (%)	3 (11%)	1 (5%)	0 (0%)	4 (5%)	0.1^*c*^
Normal karyotype, n (%)	0 (0%)	11 (61%)	4 (13%)	15 (20%)	<0.0001
Balanced translocation, n (%)	1 (4%)	0 (0%)	18 (60%)	19 (26%)	<0.0001
Complex karyotype, n (%)	21 (84%)	3 (17%)	1 (3%)	25 (34%)	<0.0001
Gene mutations, median (SD)	8 (29%)	12 (63%)	12 (40%)	32 (42%)	0.06
ELN 2017 AML Ccssification, n (%)					<0.0001
Adverse	13 (100%)	2 (33%)	6 (20%)	21 (43%)	
Favorable	0 (0%)	1 (17%)	14 (47%)	15 (31%)	
Intermediate	0 (0%)	3 (50%)	10 (33%)	13 (27%)	
MDS IPSS score, n (%)					0.03
Low/Int1	0 (0%)	4 (30%)	0 (0%)	4 (14%)	
Int2/High	15 (100%)	9 (69%)	0 (0%)	24 (86%)	
TRMN treatment, n (%)					<0.0001
Intensive	6 (21%)	3 (16%)	25 (83%)	34 (44%)	
Low dose	13 (46%)	10 (53%)	4 (13%)	27 (35%)	
BSC	9 (32%)	6 (32%)	1 (3%)	16 (21%)	
CR rate, n (%)	10 (36%)	7 (37%)	25 (83%)	42 (55%)	0.0003
HSCT, n (%)	5 (18%)	2 (11%)	9 (30%)	16 (21%)	0.2
Median OS, mo (95% CI)	7.5 (3.5–15.4)	14.5(7.5–41.5)	25.2 (13.3–NR)	13.9 (10.5–20.4)	<0.0001^*b*^
Median EFS, mo (95% CI)	6.2 (3.5–13.2)	10.6 (7.5–41.5)	18.4 (7.1–NR)	10.1 (6.8–16.3)	0.004^*b*^

^*a*^Wilcoxon test.

^*b*^Logrank test.

^*c*^Pearson's Chi-square test.

AML = acute myeloid leukemia; BSC = best supportive care; CHIP-AM = CHIP-associated mutations; CI = confidence interval; CR = complete remission; EFS = event-free survival; HSCT = hematopoietic stem cell transplantation; IQR = intervalle quartile range; MDS = myelodysplastic syndrome; NR = not reached; OS = overall survival; TRMN = therapy-related myeloid neoplasm.

### Survival analysis

**Figure 2.**
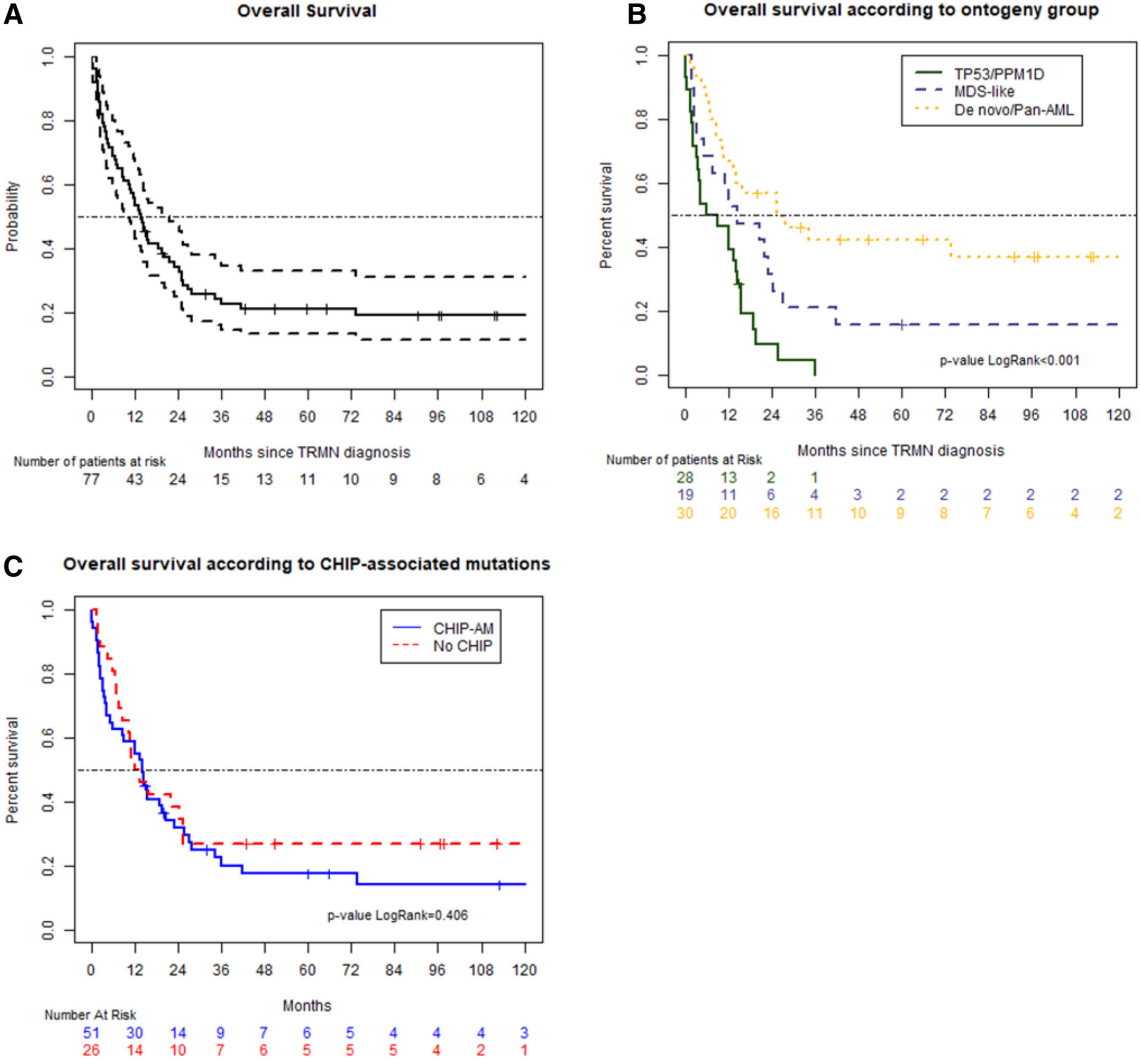
**Survival curves.** OS of the whole cohort (A), dot lines represent 95% CI. OS according modified genetic ontogeny-based classifier groups described by Lindsley et al. (B), curves show patients with “de novo/pan-AML” (yellow), “MDS-like” (blue), and “*TP53/PPM1D*” (green) mutations. OS according to CHIP-AM groups (C), blue line represents CHIP–AM patients, red line No CHIP-AM patients. AML = acute myeloid leukemia; CHIP-AM = clonal hematopoiesis of indeterminate potential-associated mutations; CI = confidence interval; MDS = myelodysplastic syndrome; OS = overall survival.

Treatment options included best supportive care for 16 patients (21%), low-dose chemotherapy for 26 patients (34%), or intensive chemotherapy for 34 patients (45%). Forty-two patients (55%) obtained a complete remission (CR) but 23 of them relapsed (55%). Sixteen patients (21%) underwent an HSCT. At time of the analysis, 17 patients were alive (22%) and 60 died (78%). With a median follow-up of 96.1 months (range: 0.1–188 mo), the median OS of the whole cohort was 13.9 months (95% CI, 10.5–20.4) (Figure 2A) and median EFS was 10.1 months (95% CI, 6.8–16.3). There was no impact of type of TRMN on OS with 13.4 months (95% CI, 8.6–34.4) and 14.4 months (95% CI, 11.2–21.9) in t-AML and t-MDS group, respectively (*P* = 0.09) (Supplemental Digital Figure 2A; http://links.lww.com/HS/A189). The number of low risk MDS was too small (n = 4) to compare OS based on IPSS classification (Supplemental Digital Figure 2C; http://links.lww.com/HS/A189). As shown in Supplemental Digital Figure 2B; http://links.lww.com/HS/A189, ELN 2017 classification correctly separated favorable AML from others but failed to discriminate intermediate and unfavorable patients.

We next evaluated impact of CHIP-AM on OS (Figure 2B). In patients bearing a TRMN with CHIP-AM, there was no impact on median OS (14.3 mo [95% CI, 8.7–23.0] versus 12.8 [95% CI, 8.6–not reached (NR)]) (Figure 2A) and EFS (9.6 mo [95% CI, 6.1–17.0] versus 10.6 [95% CI, 5.7–34.6]).

Based on the modified classifier from Lindsley et al, CR rates of “TP53/PPM1D,” “MDS-like,” and “de novo/pan-AML” were 36% (n = 10/28), 37% (n = 7/19), 83% (n = 25/30) (*P* = 0.0003). Median OS and EFS were 7.5 months (95% CI, 3.5–15.4) and 6.2 months (95% CI, 3.5–13.2) for the “TP53/PPM1D” subgroup, 14.5 (95% CI, 7.5–41.5) and 10.6 (95% CI, 7.5–41.5) for the “MDS-like” subgroup, and 25.2 (95% CI, 13.3–NR) and 18.4 (95% CI, 7.1–NR) months for the “de novo/pan-AML” subgroup, respectively (*P* < 0.001) (Figure 2C).

Median OS was 27.7 months (95% CI, 19.5–NR), 13.5 (95% CI, 10.5–18.7), and 2.85 (95% CI, 2.0–7.5) months in the intensive chemotherapy, low-dose chemotherapy, or best supportive care (BSC) group, respectively (p<0.001) (Supplemental Digital Figure 3A; http://links.lww.com/HS/A189). Median OS of patients who underwent HSCT was not reached (Supplemental Digital Figure 3B; http://links.lww.com/HS/A189).

In univariate analysis (including baseline patient characteristics), age at TRMN diagnosis (HR = 1.03, [95% CI, 1.01–1.06], *P* = 0.008), gynecological cancer (HR = 2.17 [95% CI, 1.27–3.68], *P* = 0.003), performance status ≥ 2 (HR = 4.27 [95% CI, 2.35–7.77], *P* < 0.001), the number of treatment lines received for first cancer (HR = 1.19 [95% CI, 1.04–1.36], *P* = 0.013), treatment received for TRMN (intensive versus low dose [HR = 2.99 (95% CI, 1.61–5.57)] versus BSC [HR = 6.45 (95% CI, 3.18–13.0634)], *P* < 0.001), and Lindsley’s modified classifier (“TP53/PPM1D” versus “MDS-like” [HR = 0.52 (95% CI, 0.27–0.99)], *P* = 0.045, versus “de novo/pan-AML” [HR = 0.28 (95% CI, 0.15–0.53)], *P* < 0.001) did influence OS. In multivariate analysis, baseline parameters influencing OS were age at TRMN (HR = 1.03 [95% CI, 1–1.06], *P* = 0.03), performance status (HR = 3.75 [95% CI, 2.04–6.91], *P* < 0.001) and Lindsley’s modified genetic classifier (“TP53/PPM1D” versus “MDS-like” [HR = 0.51 (0.25–1.06), *P* = 0.1, versus “de novo/pan-AML” [HR = 0.41 (95% CI, 0.2–0.82)], *P* = 0.012) (Table 3).

**Table 3.** Univariate and Multivariate Analyses for Overall Survival.

		Univariate Analysis		Multivariate Analysis	
		Crude HR (95% CI)	*P*	Adjusted HR (95% CI)	*P*
Age at TRMN		1.03 (1.01–1.06)	0.008	1.03 (1.00–1.06)	0.03
First cancer	Breast cancer	1		1	
	Gynecological cancer	2.17 (1.27–3.68)	0.004	0.97 (0.51–1.83)	0.9
Performance status	0–1	1		1	
	2–3	4.27 (2.35–7.77)	<0.001	3.75 (2.04–6.91)	<0.001
Lindsley’s modified classifier	TP53/PPM1D	1		1	
	MDS-like	0.52 (0.27–0.99)	0.045	0.51 (0.25–1.06)	0.1
	De novo/Pan-AML	0.28 (0.15–0.53)	<0.001	0.41 (0.20–0.82)	0.012
Number of treatment lines		1.19 (1.04–1.36)	0.013	1.16 (0.99–1.36)	0.1

Global logRank test *P* < 0.0001.

CI = confidence interval; TRMN = therapy-related myeloid neoplasm.

## Discussion

In this article, we uncovered the molecular landscape of TRMN with a large NGS gene panel in a cohort of gynecological and breast cancer survivors. TRMN studies are generally very heterogeneous in term of primary cancer type,^19–21^ and/or focus on a TRMN subtype such as t-AML^13^ or t-MDS.^22^ We think that our work is a good representation of TRMN in women with breast and gynecological cancers, which represent around one third of female cancer.^23^ Moreover, we combine NGS data with detailed patient characteristics to deeply understand mechanisms underlying this secondary disease.

The clinical features of our cohort are quite similar to other TRMN studies, except for a higher percentage of t-AML mainly due to the availability of genomic DNA at TRMN diagnosis (detailed Supplemental Digital Table 1; http://links.lww.com/HS/A189). Molecular results are in line with previous reports^13,19,20,24^ except for *ASXL1* mutations (5%), described in 26% of t-MDS^24^ and 17% of t-AML.^13^ We found a higher frequency of *TP53* (31%) mutations and lower frequency of *NPM1* (10%) and *FLT3* (9%) in TRMN compared to de novo AML/MDS as described by others.^13,19,21,24^ More interestingly, we described 9% of *PPM1D* mutations, a gene usually not included in myeloid NGS panels. PPM1D is a Ser/Thr protein phosphatase that negatively regulates *TP53* and affects functional DNA damage response. The emergence of *PPM1D* mutations is associated with prior exposure to specific DNA-damaging agents as it has been shown for *TP53*.^25^ Indeed, PPM1D mutations provide a survival advantage onto hematopoietic clones by rendering them resistant to apoptosis and confer to HSC resistance to chemotherapy leading to expansion during cancer treatment. The exact role in leukemogenesis is unknown, and doubt exists to know if this mutation is more a passenger or driver mutation. It has been shown in a large series of MDS patients^22^ that *PPM1D* was more present in t-MDS (14%) than de novo MDS and often co-occurs with TP53 (44%) with a median variant allele frequency (VAF) of 5%. In our cohort, 7 patients had *PPM1D* mutations, 5 of them had a complex karyotype and the 2 others had monosomy seven. VAF of *PPM1D* mutation was low in patients with TP53 mutation (2%, 8%, and 9%) but high in patients without *TP53* mutation (19%, 24%, 28%, and 41%) suggesting that his role in leukemogenesis may be considered depending on the presence of *TP53* mutation or not.

Although TRMN is recognized as a distinct entity in 2016 WHO classification of hematological malignancies,^1^ TRMN remains a very heterogeneous disease. NGS could help distinguishing different entities that should be considered separately. We first evaluated impact of “CHIP-AM” mutations. The recent discovery of CHIP in healthy individuals suggested that myeloid neoplasms may have a premalignant condition characterized by clonal hematopoiesis.^7,26^ As shown in this study and by others,^27,28^ the existence of CHIP-AM at the cancer stage is detectable in 75% of the patients. Undetectable CHIP can be due to the detection limit of the NGS assay, not efficient under 0.1%, but it gives us some confidence to extrapolate that the majority of CHIP-AM we identified in the TRMN cohort was indeed present at the cancer stage in a minor clone. “CHIP-AM” patients (66% of our cohort) were older at TRMN diagnosis and the time interval between TRMN and first cancer was longer than the “no-CHIP” patients. Patients with CHIP-AM more frequently had an MDS phenotype, a complex karyotype and less commonly a balanced translocation. Two different peaks of incidence in TRMN have been well described.^4,29^ The first one occurred with a short latency (2–3 y). This mechanism is mediated by topoisomerase 2 inhibitors, which induce a double-strand break during DNA replication and can link 2 DNA strands together after replication, leading to fusion oncogenes responsible for t-AML.^30^ The second peak occurred with a long latency, usually described as following treatment with alkylating agents and/or radiation therapy, mimicking MDS features. Interestingly, our “CHIP-AM” and “no-CHIP” categories fit with this description, supporting the idea of a preexisting clone emerging under chemotherapy or radiotherapy in TRMN with CHIP-AM. In the healthy population, most common CHIP mutations are *DNMT3A* (52%), *TET2* (9%) and *ASXL1* (8%). *TP53* and *PPM1D* are found in only 3% and 5% (Supplemental Digital Table 2; http://links.lww.com/HS/A189).^7,26^ In cancer patients, *PPM1D* and *TP53* CHIP mutations are overrepresented,^6,25,31^ especially due to exposure to both chemotherapy and radiotherapy. Moreover, *TP53* and *PPM1D* variant allele fraction rise under cancer treatment as opposed to *TET2* and *DNMT3A* mutations. Recently, 2 large studies have shown that it was possible to predict the AML risk in healthy individuals years before diagnosis, based on the detection of CHIP.^32,33^ Interestingly, *TP53*, *IDH1/2* and spliceosomal mutations (including *SRSF2* and *U2AF1*) are associated with a higher risk of subsequent AML, in contrast with other mutations such as *DNMT3A* and *TET2* mutations. Larger studies will help to clearly distinct the role of each mutation in the development of TRMN, but we can extrapolate that the presence of DNA damage response gene mutations (ie, *TP53*, *ATM*, *CHEK2*, and *PPM1D*) could be considered as a preleukemic stage increasing the risk of TRMN. By contrast, the role of previous cancer treatment in TRMN emergence in TRMN with *TET2/DNMT3A* mutations is uncertain. These findings could be an explanation for the chemoresistance of TRMN with DNA damage response mutations.

We next thought that the ontogeny-based classification proposed by Lindsley et al^13^ for AML could allow a perfectly understandable distinction of genetic subgroups. We proposed some adjustments given that MDS patients and *PPM1D* mutations were not taken into account in the study by Lindsley et al. Based on the close interaction with TP53 in DNA damage response, we decided to consider *PPM1D* in the *TP53* group more than *“*MDS-like” or “de novo/pan-AML*”group* in our Lindsley’s modified classifier, but larger series will help to clarify “prognosis role of PPM1D mutations” in the future. This classification segregates TRMN with clinical, biological, and survival differences. A “TP53/PPM1D*”* subgroup including patients with long history of cancer treatment and complex cytogenetics, a “MDS-like” subgroup with older patients, similar to standard high-risk MDS or secondary AML, and a “de novo/pan-AML” subgroup in which most patients have a balanced chromosomal translocation. These genetic subgroups correlate with OS and appear to be more efficient than morphologic distinction between t-AML and t-MDS, suggesting that the next TRMN classification would benefit from the incorporation of cytogenetic and molecular markers.

In conclusion, our study highlights the importance of genomic characterization of TRMN for prognosis as well as a proper understanding of oncogenic mechanisms. The integration of genetic features into the future TRMN classification could improve our understanding of the biological diversity of TRMN and our ability to predict clinical outcome. The most important challenge is now to improve the OS of TRMN patients. Development of news drugs such as VYXEOS, a liposomal formulation of cytarabine and daunorubicin has shown very impressive results in patients with t-AML fit for intensive chemotherapy.^34,35^ In phase 1b/2 in combination with 5-AZACYTINE, APR-246, a small molecule that selectively induces apoptosis in *TP53*-mutated cancer cells, showed promising results in unfit *TP53* mutated AML/MDS patients.^36,37^ However, much therapeutic progress has still to be made for TRMN patients.

## Disclosures

JBM received honoraria from Abbvie, Jazz Pharmaceuticals, and Astellas. SDB received honoraria from Agios, Celgene, Forma Therapeutics, Abbvie, Astellas, Daichi, Novartis, Pfizer, and Jazz Pharmaceuticals and has received research funding from Agios and Forma Therapeutics. CM received honoraria from Astellas. SD received honoraria from Pfizer, AstraZeneca, Roche Genentech and has received research funding from Novartis, Pfizer, AstraZeneca, Roche Genentech, Lilly, Puma, Myriad, Orion, Amgen, Sanofi, Genomic Health, GE, Servier, MSD, BMS, and Pierre Fabre. AL reports grants, personal fees and nonfinancial support from AZ, grants, personal fees and nonfinancial support from Tesaro, grants, personal fees and nonfinancial support from clovis, grants and personal fees from MSD, personal fees from biocad, grants and personal fees from ability, other from merck serono, personal fees from seattle genetics, grants, nonfinancial support and other from GSK, personal fees from Zentalis, outside the submitted work. ER received honoraria from BMS, Clovis, Astra Zeneca. The other authors have no conflicts of interest to disclose.

## Sources of funding

SK-H received a grant for DUERTECC/ EURONCO (Diplôme Universitaire Européen de Recherche Translationnelle Et Clinique en Cancérologie). JB Micol received a support from « Association Laurette Fugain » (grant number ALF 2019/11).

## Supplementary Material



## References

[1] Arber DA Orazi A Hasserjian R. The 2016 revision to the World Health Organization classification of myeloid neoplasms and acute leukemia. Blood. 2016;127:2391–2405. 27069254 10.1182/blood-2016-03-643544

[2] Morton LM Dores GM Schonfeld SJ. Association of chemotherapy for solid tumors with development of therapy-related myelodysplastic syndrome or acute myeloid leukemia in the modern era. JAMA Oncol. 2019;5:318–325. 30570657 10.1001/jamaoncol.2018.5625 PMC6439835

[3] Shenolikar R Durden E Meyer N. Incidence of secondary myelodysplastic syndrome (MDS) and acute myeloid leukemia (AML) in patients with ovarian or breast cancer in a real-world setting in the United States. Gynecol Oncol. 2018;151:190–195. 30268525 10.1016/j.ygyno.2018.09.003

[4] Heuser M. Therapy-related myeloid neoplasms: does knowing the origin help to guide treatment? Hematology Am Soc Hematol Educ Program. 2016;2016:24–32. 27913458 10.1182/asheducation-2016.1.24 PMC6142514

[5] McNerney ME Godley LA Le Beau MM. Therapy-related myeloid neoplasms: when genetics and environment collide. Nat Rev Cancer. 2017;17:513–527. 28835720 10.1038/nrc.2017.60 PMC5946699

[6] Bolton KL Ptashkin RN Gao T. Cancer therapy shapes the fitness landscape of clonal hematopoiesis. Nat Genet. 2020;52:1219–1226. 33106634 10.1038/s41588-020-00710-0 PMC7891089

[7] Bowman RL Busque L Levine RL. Clonal hematopoiesis and evolution to hematopoietic malignancies. Cell Stem Cell. 2018;22:157–170. 29395053 10.1016/j.stem.2018.01.011 PMC5804896

[8] Hsu JI Dayaram T Tovy A. PPM1D mutations drive clonal hematopoiesis in response to cytotoxic chemotherapy. Cell Stem Cell. 2018;23:700–713.e6. 30388424 10.1016/j.stem.2018.10.004 PMC6224657

[9] Wong TN Ramsingh G Young AL. Role of TP53 mutations in the origin and evolution of therapy-related acute myeloid leukaemia. Nature. 2015;518:552–555. 25487151 10.1038/nature13968 PMC4403236

[10] Smith SM Le Beau MM Huo D. Clinical-cytogenetic associations in 306 patients with therapy-related myelodysplasia and myeloid leukemia: the University of Chicago series. Blood. 2003;102:43–52. 12623843 10.1182/blood-2002-11-3343

[11] Ok CY Patel KP Garcia-Manero G. TP53 mutation characteristics in therapy-related myelodysplastic syndromes and acute myeloid leukemia is similar to de novo diseases. J Hematol Oncol. 2015;8:45. 25952993 10.1186/s13045-015-0139-z PMC4431603

[12] Braun T Cereja S Chevret S; French-Belgian-Swiss APL Group. Evolving characteristics and outcome of secondary acute promyelocytic leukemia (APL): a prospective analysis by the French-Belgian-Swiss APL group. Cancer. 2015;121:2393–2399. 25845577 10.1002/cncr.29389

[13] Lindsley RC Mar BG Mazzola E. Acute myeloid leukemia ontogeny is defined by distinct somatic mutations. Blood. 2015;125:1367–1376. 25550361 10.1182/blood-2014-11-610543 PMC4342352

[14] Renneville A Boissel N Gachard N. The favorable impact of CEBPA mutations in patients with acute myeloid leukemia is only observed in the absence of associated cytogenetic abnormalities and FLT3 internal duplication. Blood. 2009;113:5090–5093. 19289855 10.1182/blood-2008-12-194704

[15] Döhner H Estey E Grimwade D. Diagnosis and management of AML in adults: 2017 ELN recommendations from an international expert panel. Blood. 2017;129:424–447. 27895058 10.1182/blood-2016-08-733196 PMC5291965

[16] Greenberg P Cox C LeBeau MM. International scoring system for evaluating prognosis in myelodysplastic syndromes. Blood. 1997;89:2079–2088. 9058730

[17] Jaiswal S Fontanillas P Flannick J. Age-related clonal hematopoiesis associated with adverse outcomes. N Engl J Med. 2014;371:2488–2498. 25426837 10.1056/NEJMoa1408617 PMC4306669

[18] Genovese G Kähler AK Handsaker RE. Clonal hematopoiesis and blood-cancer risk inferred from blood DNA sequence. N Engl J Med. 2014;371:2477–2487. 25426838 10.1056/NEJMoa1409405 PMC4290021

[19] Ok CY Patel KP Garcia-Manero G. Mutational profiling of therapy-related myelodysplastic syndromes and acute myeloid leukemia by next generation sequencing, a comparison with de novo diseases. Leuk Res. 2015;39:348–354. 25573287 10.1016/j.leukres.2014.12.006 PMC5548131

[20] Shih AH Chung SS Dolezal EK. Mutational analysis of therapy-related myelodysplastic syndromes and acute myelogenous leukemia. Haematologica. 2013;98:908–912. 23349305 10.3324/haematol.2012.076729 PMC3669447

[21] Kuzmanovic T Patel BJ Sanikommu SR. Genomics of therapy-related myeloid neoplasms. Haematologica. 2020;105:e98–e101. 31413096 10.3324/haematol.2019.219352 PMC7049337

[22] Lindsley RC Saber W Mar BG. Prognostic mutations in myelodysplastic syndrome after stem-cell transplantation. N Engl J Med. 2017;376:536–547. 28177873 10.1056/NEJMoa1611604 PMC5438571

[23] Bray F Ferlay J Soerjomataram I. Global cancer statistics 2018: GLOBOCAN estimates of incidence and mortality worldwide for 36 cancers in 185 countries. CA Cancer J Clin. 2018;68:394–424. 30207593 10.3322/caac.21492

[24] Singhal D Wee LYA Kutyna MM. The mutational burden of therapy-related myeloid neoplasms is similar to primary myelodysplastic syndrome but has a distinctive distribution. Leukemia. 2019;33:2842–2853. 31089247 10.1038/s41375-019-0479-8

[25] Coombs CC Zehir A Devlin SM. Therapy-related clonal hematopoiesis in patients with non-hematologic cancers is common and associated with adverse clinical outcomes. Cell Stem Cell. 2017;21:374–382.e4. 28803919 10.1016/j.stem.2017.07.010 PMC5591073

[26] Danlos FX Papo M Micol JB. Clonal haematopoiesis: a concise review. Rev Med Interne. 2019;40:684–692. 31126662 10.1016/j.revmed.2019.05.005

[27] Gillis NK Ball M Zhang Q. Clonal haemopoiesis and therapy-related myeloid malignancies in elderly patients: a proof-of-concept, case-control study. Lancet Oncol. 2017;18:112–121. 27927582 10.1016/S1470-2045(16)30627-1 PMC7771361

[28] Takahashi K Wang F Kantarjian H. Preleukaemic clonal haemopoiesis and risk of therapy-related myeloid neoplasms: a case-control study. Lancet Oncol. 2017;18:100–111. 27923552 10.1016/S1470-2045(16)30626-X PMC5405697

[29] Jabagi MJ Vey N Goncalves A. Evaluation of the incidence of hematologic malignant neoplasms among breast cancer survivors in France. JAMA Netw Open. 2019;2:e187147. 30657534 10.1001/jamanetworkopen.2018.7147 PMC6484549

[30] Mistry AR Felix CA Whitmarsh RJ. DNA topoisomerase II in therapy-related acute promyelocytic leukemia. N Engl J Med. 2005;352:1529–1538. 15829534 10.1056/NEJMoa042715

[31] Martin JE Khalife-Hachem S Grinda T. Therapy-related myeloid neoplasms following treatment with PARP inhibitors: new molecular insights. Ann Oncol. 2021;32:1046–1048. 34107346 10.1016/j.annonc.2021.04.015

[32] Desai P Mencia-Trinchant N Savenkov O. Somatic mutations precede acute myeloid leukemia years before diagnosis. Nat Med. 2018;24:1015–1023. 29988143 10.1038/s41591-018-0081-z PMC6849383

[33] Abelson S Collord G Ng SWK. Prediction of acute myeloid leukaemia risk in healthy individuals. Nature. 2018;559:400–404. 29988082 10.1038/s41586-018-0317-6 PMC6485381

[34] Lancet JE Uy GL Cortes JE. CPX-351 (cytarabine and daunorubicin) liposome for injection versus conventional Cytarabine plus Daunorubicin in older patients with newly diagnosed secondary acute myeloid leukemia. J Clin Oncol. 2018;36:2684–2692. 30024784 10.1200/JCO.2017.77.6112 PMC6127025

[35] Chiche E Rahmé R Bertoli S. Real-life experience with CPX-351 and impact on the outcome of high-risk AML patients: a multicentric French cohort. Blood Adv. 2021;5:176–184. 33570629 10.1182/bloodadvances.2020003159 PMC7805314

[36] Cluzeau T Sebert M Rahmé R. Eprenetapopt plus Azacitidine in TP53-mutated myelodysplastic syndromes and acute myeloid leukemia: a phase II study by the Groupe Francophone des Myélodysplasies (GFM). J Clin Oncol. 2021;39:1575–1583. 33600210 10.1200/JCO.20.02342 PMC8099409

[37] Sallman DA DeZern AE Garcia-Manero G. Eprenetapopt (APR-246) and Azacitidine in TP53-mutant myelodysplastic syndromes. J Clin Oncol. 2021;39:1584–1594. 33449813 10.1200/JCO.20.02341 PMC8099410

