# Supplementary material for: Molecular Landscape of Therapy-related Myeloid Neoplasms in Patients Previously Treated for Gynecologic and Breast Cancers
Source: Hemasphere. 2021 Aug 18;5(9):e632. doi: 10.1097/HS9.0000000000000632 (PMC8373540; doi:10.1097/HS9.0000000000000632)
Supplement: Supplementary file 1 [file hs9-5-e632-s001.docx]

Supplementary materials

**NGS panel :**

NGS targeted mutational analysis using a panel of 74 genes*: ABL1, ANKRD26, APC, ASXL1, ASXL2, ATG2B, ATM, ATRX, BCOR, BCORL1, BRAF, CALR, CBL, CEBPA, CHEK2, CREBBP, CSF3R, CUX1, DDX41, DDX54, DHX29, DIS3, DNMT3A, EED, EP300, EPOR, ERBB4, ETNK1, ETV6, EZH2, FLT3, GATA1, GATA2, HRAS, IDH1, IDH2, JAK2, KDM6A, KIT, KRAS, MPL, MYC, NF1, NFE2, NPM1, NRAS, PHF6, PPM1D, PRPF40B, PRPF8, PTPN11, RAD21, RUNX1, SETBP1, SETD2, SF1, SF3A1, SF3B1, SH2B3, SMC1A, SMC3, SRP72 , SRSF2, STAG2, SUZ12, TERC, TERT, TET2, THPO, TP53, U2AF1,* *U2AF2*, *WT1*, *ZRSR2.*

**

Sup Fig 1 : Comutation plot of the 77 TRMN patients, mutations are depicted by colored bars, and each column represents 1 of the 77 sequenced subjects. Colors reflect CHIP-AM or No-CHIP groups

Sup Fig 2 : OS of the whole cohort (a), according to type of hematologic malignancy (t-AML and t-MDS) (b), AML classification (ELN 2017), MDS classification (IPSS) (c) and OS in the CHIP-AM cohort according to presence of DDR mutation.

Sup Fig 3 : OS of the whole cohort according to treatment received (a) and HSCT (b).

**Sup Fig 1**


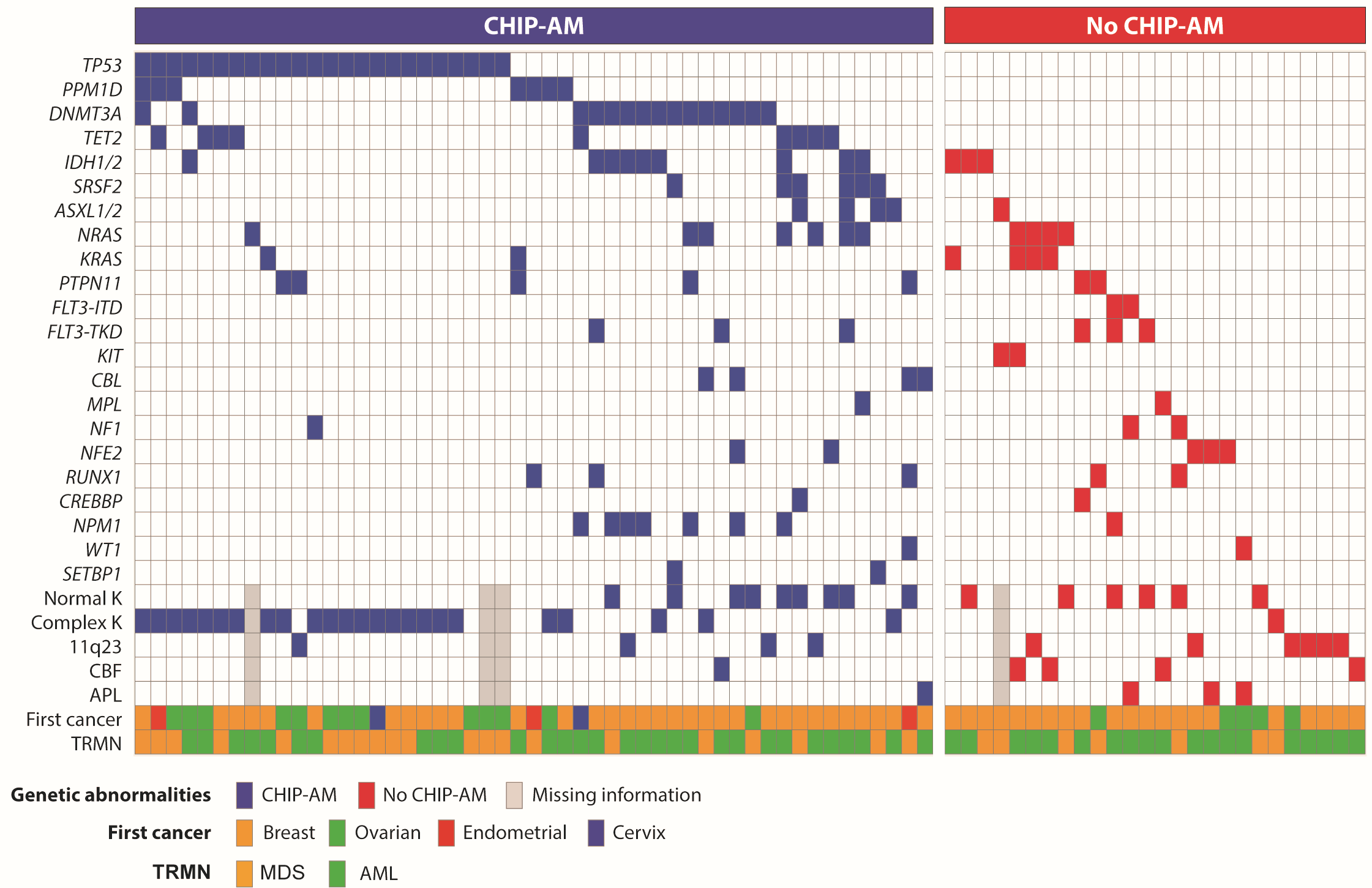


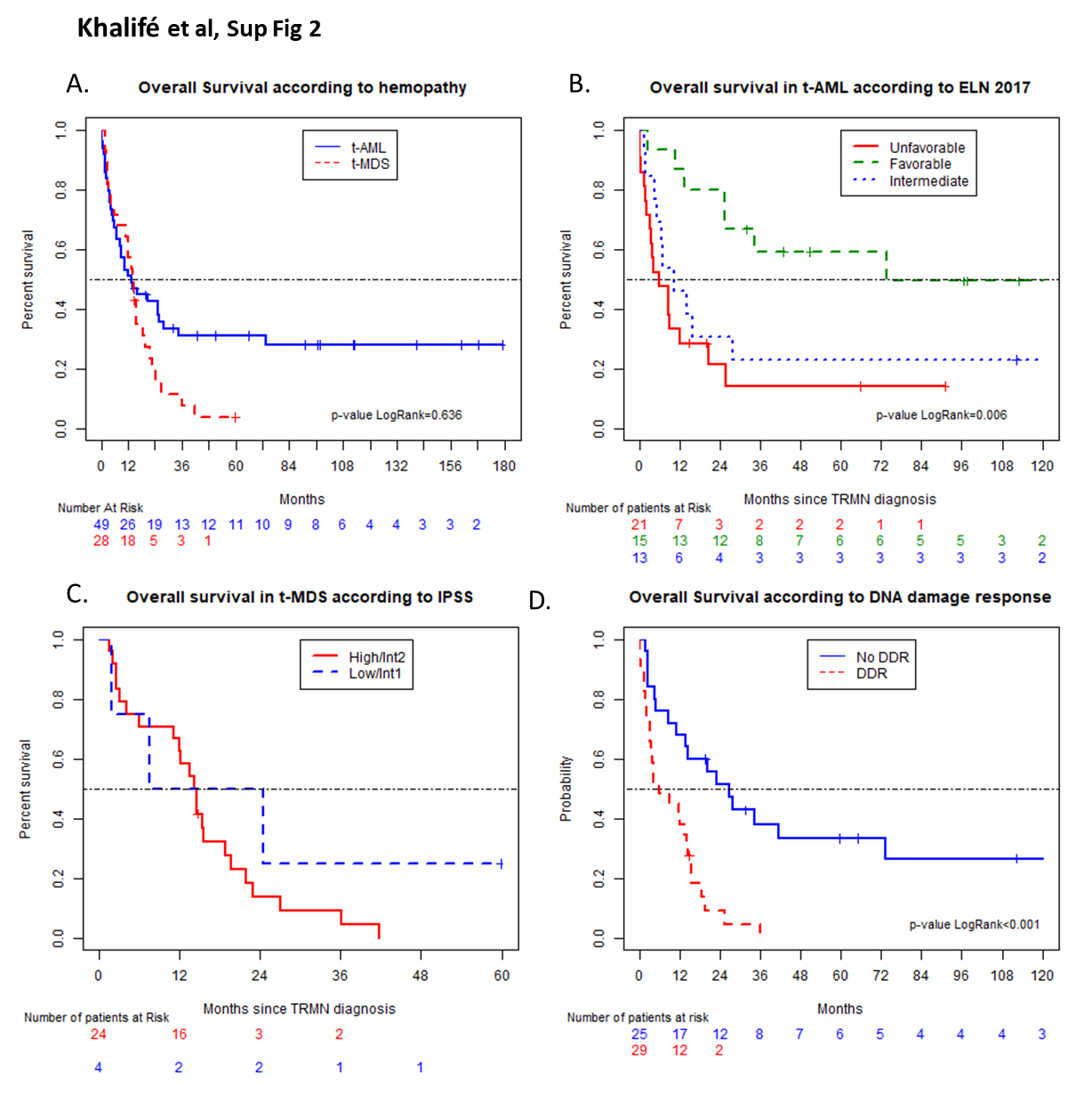


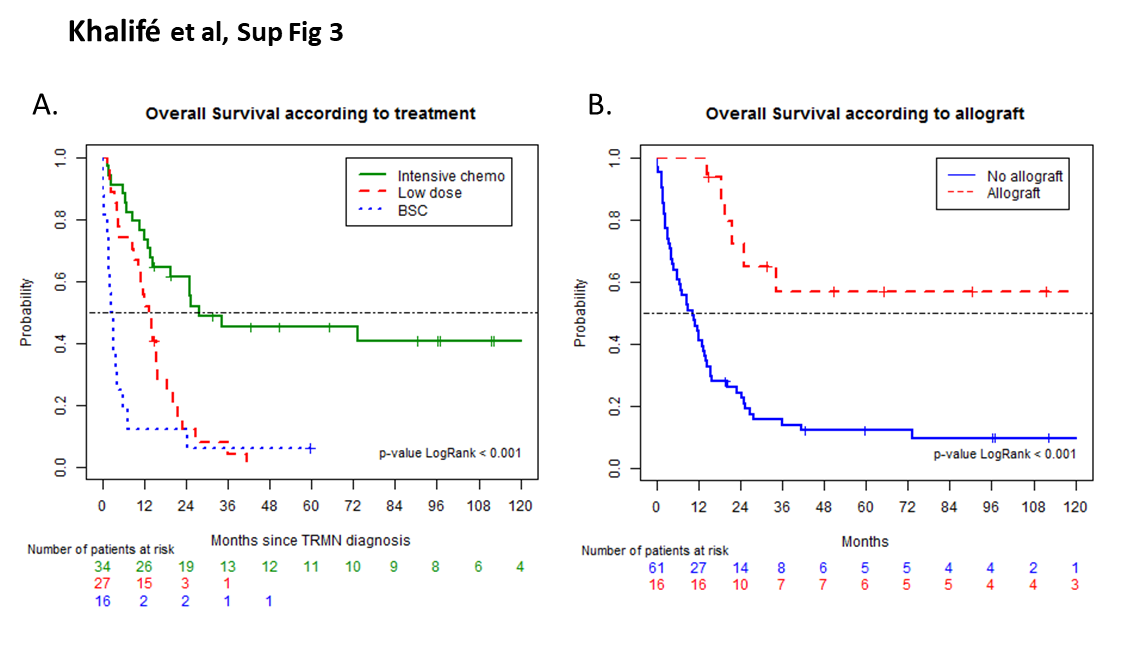


Supp Table 1: Patients treated for breast or gynecologic cancers with TRMN included in Gustave Roussy database according to DNA availability (selected patients)

Supp Table 3: NGS mutations identified in TRMN patients

Supp Table 4: Mutations and variant frequencies at time of CHIP and therapy-related myeloid neoplasm samples
